# Assessing medical students’ perception and educational experience during COVID-19 pandemic

**DOI:** 10.1007/s11845-022-03118-3

**Published:** 2022-07-30

**Authors:** Ernest Z. Low, Niall J. O’Sullivan, Vidushi Sharma, Isabella Sebastian, Roisin Meagher, Dalal Alomairi, Ebraheem H. Alhouti, Claire L. Donohoe, Michael E. Kelly

**Affiliations:** Trinity College Dublin, St. James’s Hospital, Dublin, Ireland

**Keywords:** COVID-19 pandemic, Digital education, Formative assessment, Hybrid learning, Medical education, Mental health, Summative assessment

## Abstract

**Introduction:**

The COVID-19 pandemic has significantly impacted the traditional delivery of medical education. Medical education programmes have had to cope with limitations on face-to-face learning, and accelerate the adoption of digital learning. In addition, the pandemic has potential serious implications on the psychological well-being of medical students. We aim to assess the changes in perceptions and experiences of medical students as a consequence of this pandemic.

**Methods:**

Cross-sectional survey of medical students at Trinity College Dublin (TCD) between March and April 2022 was performed. The survey explored student satisfaction with the current education program, teaching delivery and the impact of COVID-19 on education and student well-being.

**Results:**

175 medical students participated in the survey. Overall, the majority of students were happy/neutral with their medical education. 93 (53.1%) felt tutorials and problem-based learning (PBL) to be the most effective method of teaching, followed by laboratory and clinical placements in 78 participants (44.6%) and hybrid-learning in 85 participants (48.6%). There was a mixed reaction to the changes in the delivery of education brought about by the pandemic. 67 participants (40.6%) felt happy with the changes, another 64 participants (38.8%) felt neutral, whilst only 34 participants (20.6%) were unhappy. However, most participants felt the pandemic negatively impacted their mental health, with 96 participants (55.8%) reporting negative responses. 58% of participants (*n* = 102/175) reported utilising the student support services at university campus and 49% (*n* = 50) were satisfied with their services.

**Conclusion:**

Digital content and delivery confer the benefit of greater flexibility in learning, the ability to learn at one’s own pace and in a preferred environment, however lacks the advantage of bedside teaching and hands-on training. Our findings reinforce the potential advantages of online learning.

## Introduction

Medical education is ever-changing and is constantly being refined to stay up to date with contemporary teaching practices [1]. Medical school curricula must constantly adapt to changing students’ needs and adopt new education delivery and platforms [2]. As we better understand different learning and teaching strategies, coupled with the advancement and integration of new educational technology, there are boundless opportunities to improve the structure, content and delivery of medical education. Most medical schools have adopted online-learning platforms, promoting self-directed learning and forming an integral part of the teaching framework [3, 4]. In particular, self-directed learning is vital in the medical profession as contemporary medical practices require constant and continual learning of new clinical skills and knowledge, and hence there is an increased emphasis on self-directed learning in medical education so as to better prepare the medical students for lifelong learning [5–7].

The first case of COVID-19 in Ireland was confirmed in February 2020 [8]. As of April 2022 the number of cases have significantly increased [9]. In response to the immense pressure on the healthcare system, restrictions were put in place by the government, with the closure of higher education institutions for in-person lectures for over a year [10, 11].

Similar to the national reforms made within the healthcare system such as the widespread adoption of telemedicine, the COVID-19 pandemic saw major changes within medical education, as a significant portion of the curriculum delivery had to be converted to an online format [12–14]. For example, small-group tutorials had to be facilitated through online interactive meetings; didactic teaching in the form of lectures were either live-streamed or recorded, whilst practical clinical skills sessions had to be redesigned to adapt to digital learning. In many instances, learning activities had to be deferred, clinical rotations and patient exposures were reduced, whilst both formative and summative examinations were digitalised [3].

The COVID-19 pandemic had posed considerable challenges to conventional learning [3]. Overall, these changes may have serious implications on the quality of education, students’ experiences and their academic performances. In addition, such educational shift has created a stressful learning environment for both the faculty and students [15] and may have negative impacts on mental well-being. It has intensified the demand for self-directed learning and arguably increased the workload for students. Our study aims to explore students’ opinions and perceptions of these changes as a consequence of the COVID-19 pandemic. Additionally, our study aims to provide contemporary insight into current medical students’ expectations and preferences, and to highlight areas of concern including mental health and coping strategies at university medical school.

## Methods

### Study design

We conducted a cross-sectional survey to explore medical students’ experiences and perceptions at TCD School of Medicine (SOM). A qualitative survey that encompasses 15 items with four sub-sections including “overall teaching experience”, “exams and assessments”, “the impact of COVID-19 on training” and “mental health” were designed. They were formulated using a five-point Likert scale (that ranged from “extremely unhappy” to “extremely happy”), multiple-choice questions and open-ended free text responses. The qualitative survey was generated using “Google Forms”, a survey administration software. Medical students at TCD were invited to participate in the survey.

### Inclusion and exclusion criteria

All undergraduate TCD medical students that ranged from Medical Year 1 to Medical Year 5, inclusive of intercalated masters medical students, were invited to participate in the study and this amounted to more than 900 medical students in the subject pool. The inclusion criteria were active medical students studying at TCD and consented to participation. Any incomplete survey was excluded from our study. A target sample size of 30% responses per year was set.

### Participant consent

Medical students were informed that participation in the study was absolutely voluntary and there would be no penalty for refusal or withdrawal from participation. Personally identifiable information such as email addresses and student ID were not collected to ensure anonymity, and participants were not given a copy of their answers.

### Data collection and sampling

The cross-sectional qualitative survey was conducted from 2 March 2022 to 8 April 2022 over a span of 5 weeks. Digital and hard-copy surveys were circulated to medical students across medical year 1 to 5 within this time frame. The hard-copy surveys were distributed to the medical students before or after lectures through their class representatives. Data collected from hard-copy surveys were then individually processed and computed by study investigators. Digital surveys’ URL were also circulated to all medical students and the link was kept active during the 5-week time frame. The function to edit response was disallowed after submission. Individuals were also limited to only a single submission through Google sign-in feature to prevent duplicated responses.

### Data analysis

The qualitative data collected from the survey was individually processed by the study investigators and all free-text responses were thematically analysed. Thematic analysis was performed and an inductive approach was used to generate themes which would reflect the recurring views of our participants. Graphical representations of the data collected were generated using Google Sheets Software and Microsoft Excel Computer Program.

## Results

A total of 175 medical students participated in the study. This represented approximately 19.4% of the sample size (*n* = 900). The largest cohort of participants were from Medical Year 2 at 38.3% (*n* = 67), followed by Medical Year 1 at 23.4% (*n* = 41), Medical Year 3 at 16% (*n* = 28), Medical Year 4 at 12% (*n* = 21) and finally Medical Year 5 at 9.7% (*n* = 17).

### Overall findings

Approximately half of the participants were satisfied with the current medical education offered at university campus. Of the participants, 47.7% (*n* = 82) were “happy” or “extremely happy” when asked about their impression of college medical training to date, 36% of participants (*n* = 62) were neutral, whilst the remaining 16.3% (*n* = 28) participants were dissatisfied.

“Library” and “home” were rated as the preferred location to study amongst the participants, as rated by 56% (*n* = 98) and 50.9% (*n* = 89), respectively. The survey found “Tutorials and PBL” to be the most effective form of pedagogical teaching, rated by 53.1% (*n* = 93) participants, followed by “Hybrid Learning” and “Laboratory and Clinical Placements” at 48.6% (*n* = 85) and 44.6% (*n* = 78), respectively.

Fifty-eight percent (*n* = 102) of participants reported utilising student support services at university campus in the past, and amongst them, 49% (*n* = 50) were satisfied with the support services they received.

### Examination and assessment preferences

The survey data found that approximately 43% of participants (*n* = 74) preferred in-person examinations, and 40% (*n* = 69) and 17.8% (*n* = 31) of the participants favoured online examination or had no preference, respectively. The survey data also found that the majority of participants (74.9%) (*n* = 131) preferred continuous assessment over summative assessments.

### Perception on increasing digital delivery of learning due to COVID-19 pandemic

The survey data found the participants deemed digital delivery of learning to be advantageous in several ways, for example being able to re-listen to lecture recordings, having the option to learn at own pace and the flexibility to learn at preferred time and location. Conversely, disadvantages of this type of teaching included a lack of hands-on training and bedside teaching.

Of the participants, 42.9% (*n* = 75) felt that the learning resources provided by university college were inadequate, 32.6% (*n* = 57) of participants felt neutral and the remaining 24.6% (*n* = 43) were happy with current resources. Of note, only 2.9% (*n* = 5) were extremely satisfied with the amount of learning resources provided. Conversely, there were more participants who felt satisfied with the quality of teaching in hospitals and laboratory placements during the pandemic. Of the participants, 40.6% (*n* = 67) were happy with the quality of teaching, whilst only 20.6% (*n* = 34) felt otherwise. The remaining 38.8% (*n* = 64) felt neutral regarding the quality of teaching during the pandemic.

### Mental health and social well-being

More than half of the survey participants, at 55.8% (*n* = 96), felt that COVID-19 pandemic had a negative impact on their mental health whilst studying at university college. “Friends and Family Members” were rated as the most popular form of mental health support, at 54% (*n* = 95) and 39.7% (*n* = 69), respectively. Of the participants, 38.2% (*n* = 65) were unhappy with the mental health support services offered at university college, whilst 37.6% (*n* = 64) felt neutral and the remaining 24.1% (*n* = 41) rated happy or extremely happy.

There were several themes identified as the recurring obstacles students faced when attempting to strike a balance between academic and social life. Major themes include “overwhelming quantity of schoolwork”, “the lack of time to spend with friends and families outside college” and “the feeling of guilt when spending time outside of studying”. Amongst the survey participants who provided free-text responses, 21% (*n* = 27) of the free-text responses suggested the overwhelming quantity of schoolwork as the primary reason for impacting their social life:

1. “The volume of work is very overwhelming and there is not enough time to cover everything”,
2. “There are too much academic workload and pressure to have a healthy social life” and
3. “I feel like we have no time for social interaction due to the copious workload of medicine. It is very hard to balance a social life and do well in school”.

Interestingly, there was also one response that attributed the COVID pandemic as a significant obstacle to achieving a study life balance — “The studies in third year were amplified by COVID pandemic and we are getting more lectures and clinical workload compared to the previous years”.

Similarly, 21% (*n* = 28) of students indicated that the lack of available time outside of studies was a major obstacle to obtaining a work-life balance:

1. “I have to limit when I can go out; I can’t go out late during weekdays, I can’t meet up with people in campus because of hospital placements”,
2. “I do not had time to meet my friends in months” and
3. “I don’t have as much time as I would like to attend to social events or to hang out with my friends” were some examples of these responses.

7% (*n* = 10) of respondents expressed a feeling of guilt when not studying as the reason for impacting their social life; some responses included the following:

1. “I often feel guilty if I’m taking a day off studying, I barely go out and feel guilty when I do so”;
2. “Even when you can socialise, the sense of guilt of not doing work is immense”; and
3. “My studies have a major effect on my social life and cause anxiety and guilt when I do get to see my friends”.

## Discussion

Our study explored medical students’ perception of their learning experiences at university medical school before and during the COVID-19 pandemic. It highlighted the challenges that the pandemic caused in the delivery of medical education. Our study had a stronger recruitment from Medical Year 1 and 2 participants, where both years are pre-clinical and have a stronger emphasis on basic sciences and less bedside teaching then clinical teaching years. Conversely, the clinical years, which included Medical Years 3 to 5, placed a stronger emphasis on practical learning through increased patient interaction and bedside teaching, and these learning activities required very specific skillsets such as history taking, physical examination and appropriate bedside manner, all of which would have been picked up during the pre-clinical years. This has important implications as the survey data collected may be skewed towards these pre-clinical years rather than normally distributed, which would have provided a more representative picture of medical students as a whole. However, it still provides some useful insights into student perceptions. Overall, the use of “Tutorials and PBLs” are felt to be most effective method of teaching. Despite the minimal exposure to clinical placements during the recent period, “laboratory and clinical placements” were also deemed very popular, with didactic lectures being less popular.

In addition, our survey also found “hybrid learning”, a model that encompasses in-person and online teaching, to be a popular method of learning. When analysing the students’ perceptions on the advantages and disadvantages brought about by digital learning, our study observed that the flexibility offered through digital learning, facilitates students to work at their own pace, in their own environment and at a time that works “best” for them. Contemporary education programs should consider augmenting teaching to reflect these opinions, and concurrently ensure that digital learning be used to supplement contact time rather than replacing in-person learning such as bedside-teaching and hands-on training, tools highly regarded by our participants. In addition, the adoption of virtual reality (VR)-based technology could supplement the lack of one-on-one or in-person training. Recent studies have highlighted the potential benefits of VR or augmented reality (AR) learning, especially in field of clinical anatomy, endoscopy and radiology [16–19], especially where access to cadavers [20] and clinical scenarios are difficult [19].

Interestingly, our study observed that participants strongly preferred continuous assessment over summative assessment as the primary method of examination (74.9% versus 13.7%, respectively). Formative assessments are often incorporated into continuous assessments, and they serve as “assessment for learning” where evaluation and feedback are provided to guide learning trajectory, which is different from “assessment of learning” seen in summative assessments [21]. There are enormous benefits of formative assessments in helping students to learn, and inform students of their knowledge gap [22–24]. Continuous assessments also allow a spaced approach to learning which has been recognised as a learning strategy to retain gained knowledge when compared to cramming technique before summative examinations [25]. Concerns over formative assessments are that they could be extremely time consuming, and students generally have negative views about the usefulness of feedback, and it often results in increased students’ expectation for more learning materials. Given the benefits of continuous assessments and the fact that students preferred continuous assessments over summative assessments, continuous assessments should be considered in future program modifications.

On exploring the medical students’ perception regarding mental health, we observed that 55% of the participants had negative perceptions on their mental well-being during the pandemic. This finding was consistent with Michaeli et al. study which evaluated medical education and mental health outcomes from 148 medical schools across nine different countries [26]. They observed that students had concerns about both their physical and mental well-being and this is concerning as to begin with medical students already have worse mental health compared to their non-medical peers [27]. At present, there are various student support services being offered at university medical school and they include the student counselling services, student learning development and welfare teams. According to recent data, 11.4% of students have utilised the counselling services in university campus in TCD between 2020 and 2021, but concerns over an underestimation of demand has been raised [28]. Our study found that 58% of participants have engaged with student support services, with half having a satisfactory experience. This highlighted the importance to raise awareness and accessibility of these services for all students. Moving forward, there needs to be further research into the tailoring of services to meet specific training and education needs. It is likely that the impact of COVID-19 will take several years to learn from and navigate.

We acknowledge that there were several limitations to our study. One weakness pertained to the rate of recruitment which amounted to approximately 20% of the sample size, despite strong efforts to recruit student participants via involvement of each class representatives and the dissemination of the survey via multiple platforms: social media, class Facebook pages and class emails. Such data perhaps reflected a real-world student engagement rate in medical education and provided an insight to students’ perception in affecting change in their education through survey participation. Another weakness was also identified in the design of our study. This study focused on Kirkpatrick’s lowest level of evaluation, that is “Reaction”, where we assessed students’ perception and level of satisfaction towards their medical training rather than evaluating the effectiveness of different pedagogies deemed as higher levels of Kirkpatrick’s evaluation [29]. Nevertheless, assessing student perceptions is an important component in evaluating medical curricular delivery, and the information drawn is critical to guide future curricular trajectory.

## Conclusion

Our study found mixed responses amongst medical students in relation to the educational shift brought about by the pandemic. Digital content and delivery offer greater flexibility in learning, with the ability to learn at one’s own pace and preferred environment but lacks bedside teaching and hands-on training. Our study highlights the popularity of continuous over summative assessments and underscores the importance of mental health support services and networks for students.
